# Angiopoietin-2 concentration in serum is associated with severe asthma phenotype

**DOI:** 10.1186/s13223-016-0112-6

**Published:** 2016-03-01

**Authors:** Joanna S. Makowska, Małgorzata Cieślak, Marzanna Jarzębska, Anna Lewandowska-Polak, Marek L. Kowalski

**Affiliations:** Department of Rheumatology, Chair of Clinical Immunology and Microbiology, Healthy Aging Research Center, Medical University of Lodz, 251 Pomorska Str, 92-213 Lodz, Poland; Department of Immunology, Rheumatology and Allergy, Medical University of Lodz, Lodz, Poland; Department of Immunology, Rheumatology and Allergy, Chair of Clinical Immunology and Microbiology, Healthy Aging Research Center, Medical University of Lodz, Lodz, Poland

**Keywords:** Severe asthma, Pathogenesis, Angiopoietins

## Abstract

**Background:**

Several proangiogenic molecules have been implicated in the pathogenies of asthmatic inflammation and remodeling. The aim of the study was to compare the concentration of proangiogenic factors in the sera of asthmatic patients and in healthy subjects (HS), and to refer the concentrations to both clinical and inflammatory markers of the disease severity.

**Methods:**

Serum was collected from 45 patients with severe/refractory asthma (SRA) and 51 patients with non-severe asthma (nSA). The control group included 30 HS. Serum concentrations of Angiopoietin-1, Angiopoietin-2, vascular endothelial growth factor (VEGF) and osteopontin were assessed by the enzyme-linked immunosorbent assay.

**Results:**

The levels of Angiopoietin-1 (68.8 ± 2.7 vs 56.4 ± 9.3 ng/ml; p < 0.05), Angiopoietin-2 (4.9 ± 0.35 vs 1.38 ± 0.14 ng/ml; p < 0.0001) and VEGF were significantly higher in asthmatic patients (n = 94) as compared to HS (255 ± 45.4 vs 424.5 ± 27.8 pg/ml; p < 0.01). The mean serum level of Angiopoietin-2 was found to be significantly higher in patients with SRA as compared to nSA patients (6.04 ± 0.46 vs 3.84 ± 0.43; p < 0.001). Angiopoietin-2 serum level correlated with respiratory function and with parameters of asthma severity: the mean number of asthma exacerbations in the preceding 12 months (R = 0.21; p < 0.05), mean number of emergency visits due to severe asthma exacerbation (R = 0.24; p < 0.04) and mean number of hospitalizations (R = 0.21; p < 0.05) or dose of inhaled glucocorticosteroids taken by the patients (R = 0.36; p < 0.001).

**Conclusion:**

Angiopoietin-2 seems to be a crucial proangiogenic cytokine overproduced in patients with SRA characterized by repeated exacerbations and Angiopoietin-2 serum levels can serve as a biomarker of severe asthma.

## Background

Asthma has been defined as ‘‘a common chronic disorder of the airways that is complex and characterized by variable and recurring symptoms, airflow obstruction, bronchial hyperresponsiveness, and an underlying inflammation” [1]. Bronchial tissue remodeling, although not included in the definition, is another key feature of asthmatic airways and has been shown to correlate with the disease severity and contribute to the airway irreversibility [2]. Remodeling of microvasculature [3, 4] and angiogenesis, which is the growth of new blood vessels from existing vessels [4, 5] in the airways, are perceived to be the key features of the tissue remodeling in patients with asthma [4, 6, 7].

Angiogenesis is a process of wound healing and organ development taking place throughout life, and the mechanisms responsible for the formation of new vessels have been extensively studied [8–10]. The mechanisms associated with angiogenesis and airway remodeling are poorly understood but seem to be regulated by the balance between proangiogenic growth factors and angiostatic proteins [11]. In chronic inflammation associated with asthma the domination of proangiogenic factors and other mediators with proangiogenic properties have been observed [12]. Angiopoietins (Angiopoietin 1 and Angiopoietin 2) and vascular endothelial growth factor (VEGF) are considered to play a leading role in angiogenesis, acting together to form new blood vessels [13]. It has been demonstrated that concentrations of angiopoietins are increased in induced sputum of asthmatic patients [14] and correlate with severity of exercise-induced asthma [15]. In one study, involving a small group of subjects elevated serum angiopoietins levels were found in asthma patients and have been associated with decreased lung function [16], suggesting that the serum level of proangiogenic factors may reflect ongoing asthmatic inflammation and serve as a potential biomarker of the disease severity. Osteopontin is another proangiogenic factor, which has been associated with the remodeling and its subepithelial expression was correlated with asthma severity [17].

The aim of the study was to compare serum levels of proangiogenic factors: Angiopoietin-1, Angiopoietin-2, VEGF and osteopontin in asthmatic patients and in healthy subjects (HS), and to refer their concentrations to the severity of asthma, clinical asthma phenotypes and inflammatory markers in serum.

## Methods

### Subjects

Sera were collected from 96 well characterized patients with bronchial asthma (mean age 45 ± 1.7 year, 38 males and 58 females) recruited from the Asthma Clinic, Department of Immunology, Rheumatology and Allergy, Medical University of Lodz. Based on American Thoracic Society (ATS) Workshop 2000 definitions asthmatics with severe/refractory asthma (SRA) were distinguished [18]. Patients not fulfilling ATS criteria were referred to as non-severe asthma (nSA). Control group included 30 nonatopic HS selected from the random sample of Lodz inhabitants (mean age 33.1 ± 1.5 year; 12 men and 18 women) (from Ga2len SARI study [19]). Both groups were not different with respect to sex and rate of smokers. Table 1 presents clinical characteristic of asthmatics and control subjects.

**Table 1:** Characteristics asthmatic patients’

	Non severe asthma	Severe/refractory asthma	Statistical significance (p)
Number of patients	51	45	
Age (years)	39.82 ± 2	51.13 ± 2.1	p < 0.01
Female/male	27/24	31/14	Ns
Atopy (%)	43 (84 %)	37 (82 %)	Ns
Duration of asthma (years)	11.7 ± 1.4	21.7 ± 1.8	Ns
Aspirin hypersensitivity (%)	12 (24 %)	12 (27 %)	Ns
Nasal polyps	15 (29 %)	11 (24 %)	Ns
BMI	24.8 ± 0.58	27.60 ± 0.7	Ns
FEV_1_ (% of predicted)	99.7 ± 2.06	69.8 ± 2.9	p < 0.05
FEV_1_/FVC %	78.1 ± 1.4	61.4 ± 1.9	p < 0.001
MEF 25-75 (% of predicted)	83.9 ± 4	38.9 ± 2.8	p < 0.001
FEV_1_ reversibility (%)	8.41 ± 0.99	21.7 ± 2	P < 0.001
Mean dose inhaled GCS (µg budesonide)	617 ± 35	2006 ± 42	p < 0.001
No of patients on oral GCS	0	26 (58 %)	p < 0.001
Mean dose of oral GCS (mg prednisone per day)	0	4.24 ± 0.7	p < 0.001
Mean number of exacerbation during 12 months	0.51 ± 0.08	1.5 ± 0.12	p < 0.001
Mean number of interventions of emergency service	0	0.18 ± 0.05	p < 0.001
Mean number of hospitalizations during last 12 months	0	0.17 ± 0.05	p < 0.001
ECP (µg/l)	9.59 ± 1.2	15.78 ± 2.6	p = 0.04
Total IgE (kU/l)	200 ± 33.9	348 ± 72.7	p = 0.03

Data presented as mean ± SEM

*BMI* body mass index, *FEV*_*1*_ forced expiratory volume in 1 s, *FVC* forced vital capacity, *GCS* glucocorticosteroids, *MEF* maximum expiratory flow

### Ethics, consent and permission

The study was approved by local Ethical committee and all patients signed informed consent to the study.

### Allergy skin testing and phenotypic characteristics

Atopy was determined based on the positive result of at least one skin prick test. In all patients skin prick tests were performed (Allergopharma, Reinbeck, Germany) and a wheal diameter of 3 mm or more in excess of the negative control was considered a positive result. The following allergens were tested: *D. pteronyssinus*, *D. farinae*, cat, dog, trees, grass mix, weeds, *Alternaria tenuis*. Aspirin hypersensitivity was diagnosed based on convincing history of adverse reaction to non-steroidal anti-inflammatory drugs (NSAIDs) and confirmed by a bronchial challenge with lysine aspirin [20]. Body mass index (BMI) was calculated as weight (kg)/height (m^2^).

### Respiratory function assessment

Respiratory function (flow-volume curve: FEV_1_, FEV_1_/FVC, MEF 25-75) was measured with an automatic spirometer (ABC Pneumo 2000RS, Poland) when patients were without inhaled short acting b_2_ agonists for at least 8 h and long acting b_2_ agonists for at least 12 h. Airway reversibility was assessed 15 min after nebulization of 2.5 mg of albuterol (Sterineb Salamol, Norton, Poland). The results were expressed as the best measurement and maximal % change from baseline.

### Serum mediator measurements

Concentrations of Angiopoietin-1 (sensitivity 10.3 pg/ml), Angiopoietin-2 (sensitivity 21.3 pg/ml), VEGF (sensitivity 9 pg/ml) and osteopontin (sensitivity 0.024 ng/ml) were assessed by enzyme-linked immunosorbent assay (ELISA: R&D; Minneapolis, USA). Total IgE, Eosinophil cationic protein (ECP) were measured in serum with ImmunoCAP system (*Pharmacia diagnostic, Sweden)*.

### Statistical analysis

Data are presented as mean ± SEMs (standard errors) or ±SD (standard deviation) as indicated. The data distribution was tested by Kolmogorov–Smirnov test. The following statistical methods were applied as appropriate and as indicated in the text: Spearman test to assess correlation, exact Fisher test, analysis of variance Kruskal–Wallis followed by post hoc Dunn’s multiple comparison test. Models of logistic regression were applied to assess the risk of SRA. p values <0.05 were considered to be significant.

## Results

### Phenotypic heterogeneity of asthmatic patients

Based on the ATS criteria 45 patients were diagnosed to have SRA and the remaining 51 patients were assigned to the non-severe (nSA) asthma group (mild and moderate severity based on the GINA criteria) (Table 1). Patients in SRA group were on average older and tended to have longer asthma duration. However, both groups did not differ with regard to atopy status, presence of nasal polyps, aspirin hypersensitivity or obesity. The mean number of exacerbations was significantly higher in SRA group, and only patients belonging to this group reported interventions of emergency service and/or hospitalizations during preceding 12 months, despite significantly higher mean dose of inhaled corticosteroids and the fact that majority (58 %) of them had received oral glucocorticosteroids. Patients with SRA had lower lung function as compared to non-severe asthmatics. Reversibility of the airway obstruction after inhalation 2.5 mg of salbutamol was significantly higher in SRA as compared to nSA (Table 1).

### Proangiogenic factors in serum from patient with asthma as compared to healthy controls

The mean levels of Angiopoietin-2 (4.9 ± 0.35 vs 1.38 ± 0.14 ng/ml; p < 0.0001), Angiopoietin-1 (68.8 ± 2.7 vs 56.4 ± 9.3 ng/ml; p < 0.05) and VEGF (255 ± 45.4 vs 424.5 ± 27.8 pg/ml; p < 0.01) were significantly higher in asthmatic patients as compared to HS. However, the mean osteopontin levels were significantly lower as compared to controls (32.5 ± 2.1 vs 47.9 ± 7.15 ng/ml; p < 0.05).

### Association of Angiopoietin-2 with severe asthma phenotype

**Fig. 1:**
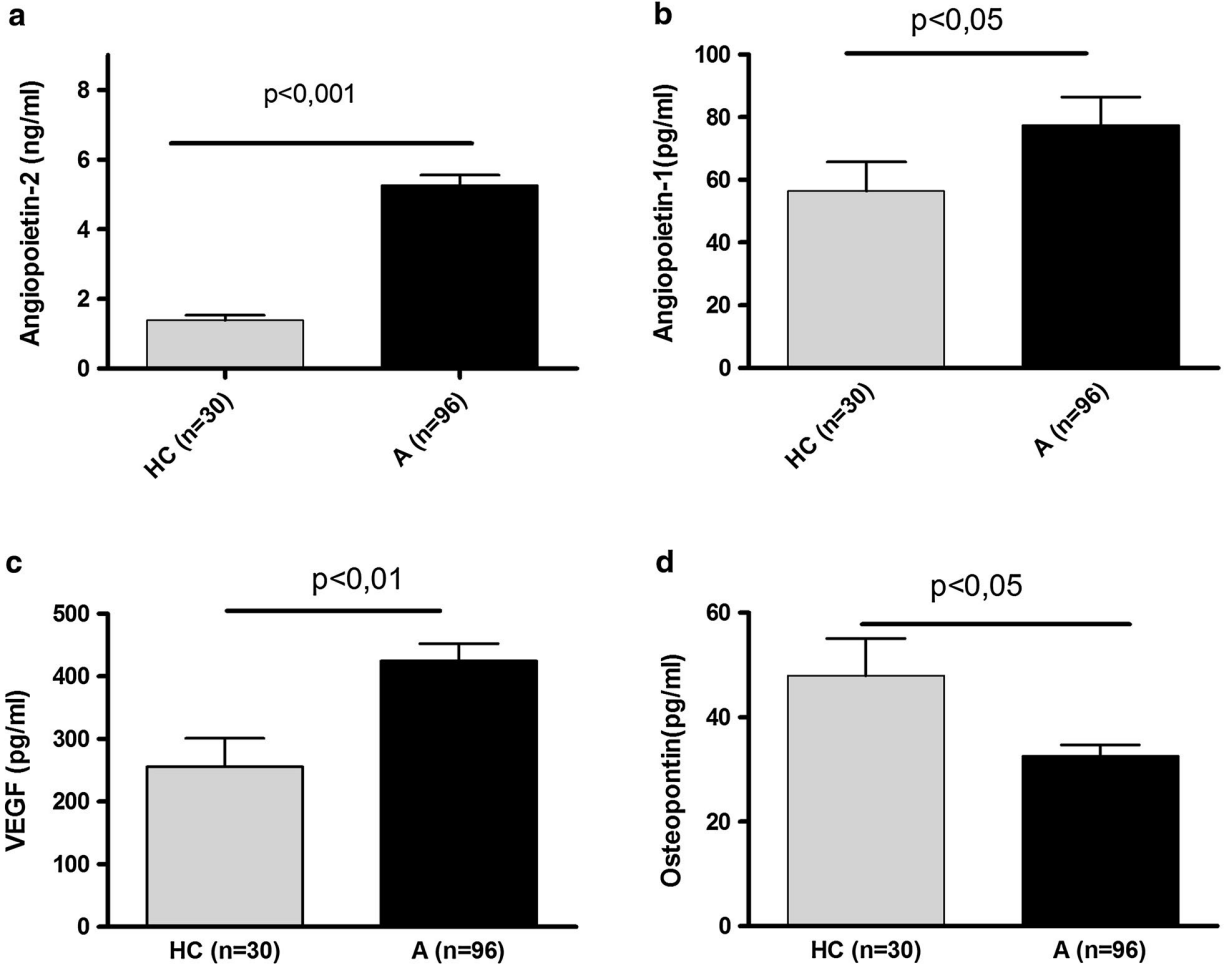
The mean (±SEM) serum levels of angiogenic factors (**a** Angiopoietin-2, **b** Angiopoietin-2, **c** VEGF, **d** Osteopontin) in patients with severe asthma (SRA), non-severe asthma (nSA) and in healthy controls (HC)

When asthmatics were stratified according to the severity, the mean serum level of Angiopoietin-2 was found to be significantly higher in patients with SRA as compared to nSA (6.04 ± 0.46 vs 3.84 ± 0.43; p < 0001), and in both asthma groups mean Angiopoietin-2 levels were higher as compared to HC (Fig. 1a). The serum levels of Angiopoietin-1, VEGF and osteopontin did not differ significantly between asthmatic subgroups (Fig. 1 b–d), but the mean Angiopoietin-1 and VGEF levels were increase in both asthma groups as compared to HC.

**Fig. 2:**
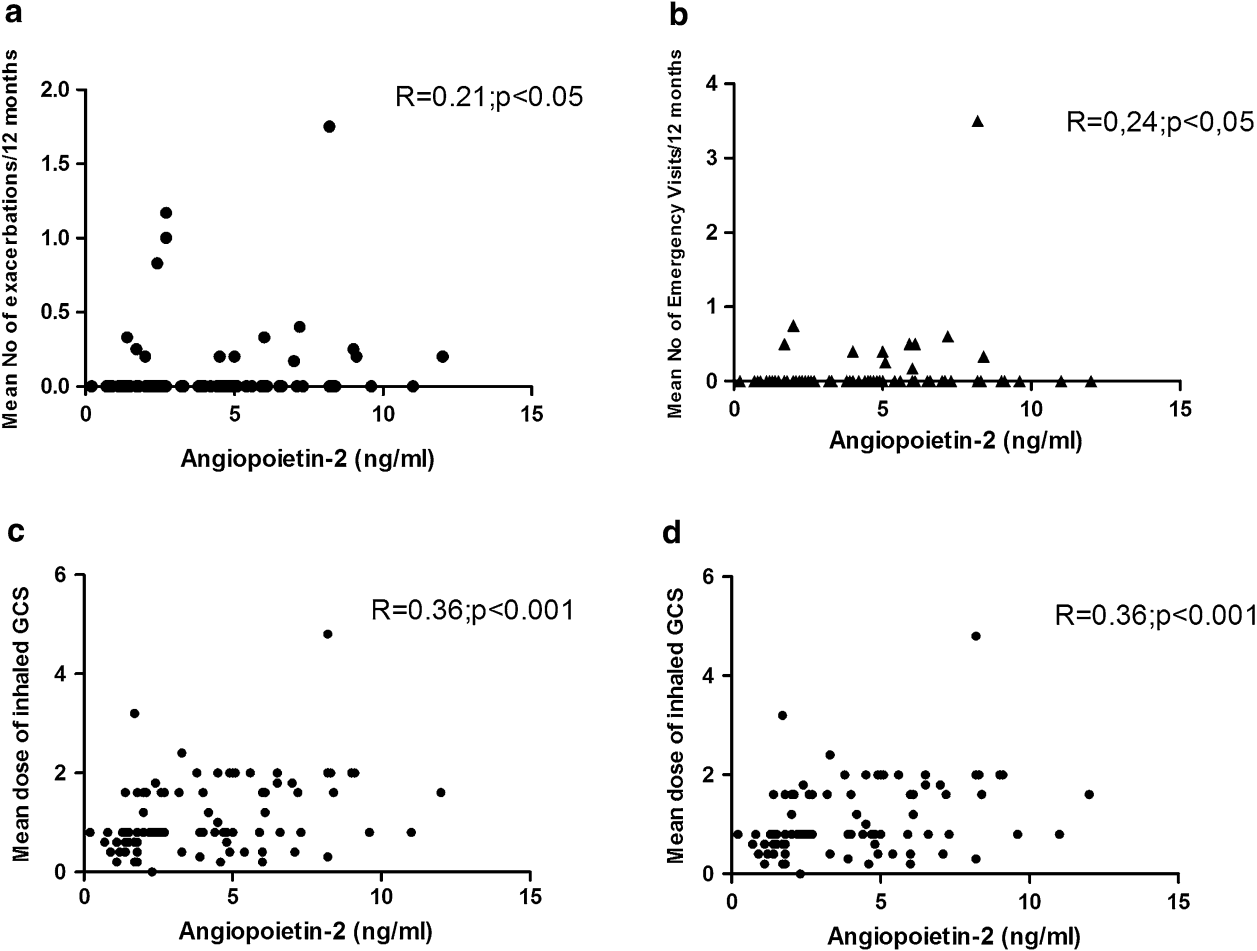
Correlation of Angiopoietin-2 serum levels with **a** number of emergency visits, **b** mean number of exacerbations, **c** mean number of hospitalizations due to asthma during last 12 months in 96 patients with asthma (Spearman rank test), **d** mean dose of inhaled glucocorticosteroids (mg of budesonide)

For the whole asthma group a modest correlation between Angiopoietin-2 serum levels and number of asthma exacerbations was found (R = 0.21; p < 0.05) (Fig. 2a), and among patients with SRA Angiopoietin-2 levels correlated with the number of emergency visits due to severe asthma exacerbation (R = 0.24; p < 0.04) (Fig. 2b) and with the number of hospitalizations (R = 0.21; p < 0.05) (Fig. 2c). There was also a significant correlation of Angiopoietin-2 serum level with the chronic dose of inhaled glucocorticosteroids taken by asthmatic patients (R = 0.36; p < 0.001) (Fig. 2d).

For the whole asthma group inverse correlation of serum Angiopoietin-2 levels with respiratory function parameters: FEV_1_% of predicted value (R = −0.31; p < 0.05) FEV_1_/FVC value (R = −0.21; p < 0.05) and MEF 25-75 (R = −0.27; p < 0.01) was found. Angiopoietin-2 correlated also with airway obstruction reversibility defined as percentage of increase in FEV_1_ after inhalation of 2.5 mg of salbutamol (R = 0.22; p < 0.05). Angiopoietin-2 correlated also with BMI of patients (R = 0.33; p < 0.001) and duration of the asthma (R = 0.2; p < 0.05).

### Proangiogenic factors in serum and other asthma clinical phenotypes

In the whole asthma group (n = 96) we did not find any association of Angiopoietin-2 or other angiogenic factors with patients’ atopic status, presence of aspirin hypersensitivity, BMI or smoking status. However, within SRA group in patients with aspirin hypersensitivity (n = 12) the mean VEGF serum levels were significantly higher as compared to aspirin tolerant individuals (n = 33; 543 ± 54 vs 369.2 ± 90 pg/ml; p < 0.05).

### Proangiogenic factors and laboratory markers of asthma severity

Serum concentrations of Angiopoietin-2, VEGF, Angiopoietin-1 and osteopontin did not correlate with classical markers of asthma severity or asthmatic inflammation: blood eosinophil number and serum ECP or total IgE levels. Significant correlations were found between Angiopoetin-2 and osteopontin (R = 0.32; p < 0.01), Angiopoietin-1 and osteopontin (R = 0.29; p < 0.01) or Angiopoietin-1 and VEGF (R = 0.25; p < 0.05) levels.

## Discussion

In this study involving relatively large and heterogeneous group of patients with asthma we documented that serum levels of Angiopoietin-2, were not only significantly increased in the asthmatics as compared to the healthy control subjects randomly selected from the general population, but also were associated with severe refractory asthma phenotype. Patients with asthma, regardless of the diseases severity had on average almost threefold higher mean Angiopoietin-2 serum levels and significantly increased Angiopoietin-1 and VEGF serum concentrations, reflecting probably ongoing angiogenesis in the airways.

We were able to distinguish a subpopulation of the asthma patients who despite high doses of oral and inhaled glucocorticosteroids suffered from severe asthma with low pulmonary function and frequent exacerbations. These patients, who fulfilled the ATS criteria for SRA, had significantly increased Angiopoietin-2 levels as compared to the patients with mild/moderate asthma. Moreover, Angiopoietin-2 serum levels correlated with several clinical features of asthma severity and control including chronic dose of inhaled glucocorticosteroids, number of asthma exacerbations, emergency room interventions and even number of hospitalizations during the preceding year. Our results are in line with an earlier report of increased levels of both Angiopoietin-1 and 2 [21, 22] in the induced sputum of severe asthmatics, which however did not correlate with the lung function tests result. In our study the increased Angiopoietin-2 concentrations constituted a significant risk factors for the severe asthma comparable to total IgE or ECP levels in serum, which are well documented asthma severity markers. However, no direct correlation of the Angiopoietin-2 levels with blood eosinophilia, serum ECP or total IgE levels was found. Other proangiogenic factors VEGF, Angiopoietin-1 and osteopontin did not correlate with asthma severity or severity serum markers.

We were able to demonstrate modest, but significant inverse correlation between the Angiopoietin-2 serum levels and FEV_1_, MEF 25-75 of predicted value, which suggests that local generation of this proangiogenic factor may have an effect on asthmatic airways function. A positive correlation found between Angiopoietin-1 levels and airway obstruction reversibility, may reflect involvement of this factor in the modulation of a smooth muscle constriction.

In an earlier study Angiopoietin-2 rather than Angiopoietin-1 was associated with asthma and Angiopoietin-1/Angiopoietin-2 ratio was positively correlated with lung function in adult asthma patients [16]. More recently, Angiopoietin-1 levels and Angiopoietin-1/Angiopoietin-2 ratio were shown to be reduced in children with asthma and no correlation of proangiogenic factors with respiratory function was found [23]. Interestingly, Angiopoietin-1 and Angiopoietin-2 levels were recently detected to be upregulated in the induced sputum from patients with optimally treated asthma, and seemed to be also related to the smoking status [22]. These not fully consistent observations may reflect differences in asthma phenotypes studied or be related to environmental influences and require further studies.

We found positive association of Angiopoietin-2 levels with asthma exacerbation defined as unscheduled visits in the clinic, emergency interventions or hospitalization. Although etiology of asthma exacerbation is multifactorial, the most common triggers are related to the respiratory infections. It has been previously demonstrated that rhinovirus infection can stimulate release of VEGF, although it had no impact on angiopoietin levels [24, 25]. Interestingly chronic bacterial infections were shown to stimulate production of Angiopoietin-2 [26]. Thus, it is likely that the association of increased serum levels of Angiopoietin-2 with the rate of asthma exacerbation may reflect a direct or non-direct release of proangiogenic factors in the airways by respiratory pathogens.

Angiopoietins (both Angiopoietin-1 and 2), which are involved in a new vessels formation in various organs are small molecules released from various cells. In addition, acting through specific Tie-2 receptor which is expressed not only on endothelial cells, but also on eosinophils and neutrophils [27, 28] angiopoietins may affect development of inflammation [27]. Neovascularization have been postulated to be one of the features of airway remodeling and both Angiopoietin-1 and Angiopoietin-2 have been already implicated in the pathogenesis of bronchial asthma [21]. Angiopoietin-1 expression is increased in the asthmatic airways. Moreover, Angiopoietin-1 reduces production of inflammatory mediators like interleukin 5 and interleukin 13, thus may have an anti-inflammatory effect in the airways [29, 20]. In contrast, Angiopoietin-2 seems to be involved in development of airway inflammation and remodeling and causes inflammation in vivo by promoting vascular leakage [31]. Thus, associations of serum angiopoietin with asthma severity markers seem to reflect involvement of proangiogenic factors in the airway inflammation and remodeling related to asthma pathogenesis and severity.

In our study proangiogenic factors other than Angiopoietin-2, did not correlate so well with asthma phenotypes. Serum levels of VEGF were higher in asthmatics as compared to HS, but no correlation with the disease severity was found. Interestingly in our study VEGF was significantly increased in patients with severe asthma and coexisting aspirin hypersensitivity. Patients with aspirin exacerbated respiratory diseases (AERD) usually have more severe asthma and were shown to have more pronounced airway remodeling [32]. It has been previously demonstrated that galectin-10 mRNA is overexpressed in peripheral blood leukocytes of AERD patients as compared to aspirin tolerant asthmatics. Galectin in turn has been linked to angiogenesis through increasing VEGF production by platelets [33, 34], thus one can speculate the increased VEGF levels found in AERD patients may reflect increased ongoing angiogenesis linked to with galectin overexpression.

Our study have confirmed and extended previous observations suggesting an imbalance between various proangiogenic factors in patients with bronchial asthma. Increased serum levels of proangiogenic Angiopoietin-2 in asthmatics and its association with clinical markers of asthma severity, including exacerbations, may reflect ongoing airway inflammation. Further studies are required to assess if Angiopoietin-2 serum level can serve as a biomarker of asthma severity and remodeling.

## Abbreviations

SRA: severe/refractory asthma
nSA: non severe asthma
VEGF: vascular endothelial growth factor
ECP: eosinophil cationic protein
NSAIDs: non-steroidal anti-inflammatory drugs
BMI: body mass index
HS: healthy subjects
